# Preliminary Study to Evaluate Three Different Treatments on Stable Chronic Obstructive Pulmonary Disease Patients Based on Markov Model

**DOI:** 10.1155/2019/6478926

**Published:** 2019-04-17

**Authors:** Xue-qing Yu, Shu-guang Yang, Han Li, Yang Xie, Jian-sheng Li, Pan Zhang

**Affiliations:** ^1^Department of Respiratory Diseases, the First Affiliated Hospital of Henan University of Chinese Medicine, Ren-min Road 19, Zhengzhou, Henan 450000, China; ^2^Collaborative Innovation Center for Respiratory Disease Diagnosis and Treatment & Chinese Medicine Development of Henan Province, Henan University of Chinese Medicine, Zhengzhou, Henan 450046, China; ^3^Henan Key Laboratory of Chinese Medicine for Respiratory Disease, Henan University of Chinese Medicine, Zhengzhou, Henan 450046, China; ^4^Henan University of Chinese Medicine, Jin-shui East Road 156, Zhengzhou, Henan 450046, China; ^5^Department of Critical Care Medicine, The Third People's Hospital of Henan Province, Fu-niu Road 198, Zhengzhou, Henan 450006, China

## Abstract

This study evaluates the costs and utilities of different treatment strategies for stable chronic obstructive pulmonary disease (COPD) patients based on Markov model and provides guidance for clinical decision and health policy making. Patients with stable COPD from four subcenters had been investigated. A Markov model with three states, namely, GOLD 1-2, GOLD 3-4, and death, was built using TreeAge Pro 2011 software. Cost-utility ratio (CUR) and incremental cost-utility ratio (ICUR) from forty Markov circles were applied to measuring the economics evaluation of three different treatments. A total of 236 stable COPD patients were randomly assigned into three groups, Western medicine group (79 cases), traditional Chinese medicine (TCM) group (79 cases), and combined group (78 cases). The results of Markov cohort simulation showed that the accumulative quality-adjusted life years (QALYs) of the three above groups per 100 000 people in 40 years were 1 702 773, 1 616 797, and 1 709 668 years, respectively, and the accumulative costs were 13 582 138 466, 1 207 904 113, and 14 656 607 371 Yuan, respectively. The CURs of the three groups were 87 235, 74 602, and 87 223 Yuan/QALY, respectively. ICURs of combined group were 8 707 and 41 705 Yuan as against Western medicine group and TCM group, respectively. Therefore, combined treatment has a lower cost, higher health output, and more socioeconomic benefits in the long run. Markov model is recommended to conduct health economics evaluation of different treatments for COPD.

## 1. Introduction

Chronic obstructive pulmonary disease (COPD) is a critical, chronic, and progressive disease with high morbidity, high mortality, and high disability rate [1]. It is characterized by persistent respiratory symptoms and airflow limitation. The most common respiratory symptoms of this disease include cough, sputum production, and shortness of breath or dyspnea, which would affect the quality of life in COPD patients seriously [2]. According to the latest data, the number of COPD patients aged 20 years or older was 99.9 million in China [1]. Annual direct costs of 204.48 billion Yuan and indirect costs of 34.10 billion Yuan were estimated to be spent on COPD [3], and hospitalization mainly caused by acute exacerbation (AE) of COPD accounted for 56.7% of the total costs, which led to a heavy socioeconomic burden. An increasingly public health problem caused by COPD has emerged. The situation is also arising in various countries all over the world [4].

Although it has serious impact on people's health, COPD can be prevented and treated. Western medicine usually adopts the comprehensive grading and staging treatment strategies recommended by Global Initiative for Chronic Obstructive Lung Disease (GOLD) [2] and Chinese Thoracic Society (CTS) [5]. AE usually causes worsening symptoms, rapid disease progression, an increased decline of lung function, and even death, so the treatment in the stable stage, which could reduce exacerbations and improve quality of life, seems to be more important for COPD patients. As the GOLD 2018 report [2] states, the main treatment goals for stable COPD are to reduce symptoms and risk of future exacerbations, among which the pharmacologic treatment makes great contribution, and the medication, such as short-acting or long-acting bronchodilator, glucocorticoid, and phosphodiesterase-4 inhibitors, should be used alone or in combination with others. A large number of clinical trials, such as TORCH [6], UPLIFT [7], INSPIRE [8], and small-dose-theophylline [9], have all confirmed that Western medicine could decrease the mortality rate and the risks of AE for stable COPD patients and slow down the annual decline of lung function. Recent study has also shown that treating patients with stable COPD is positive for managing the disease and preventing its exacerbation [10] and progression [11]. The medication can alleviate the clinical symptoms of COPD, but it cannot stop or reverse the decline of lung function, and some important problems caused by Western medicine need to be solved, such as side effects of medicine and pneumonia [6, 8].

TCM has a long history and certain efficacy in the treatment for COPD, especially in the stable stage. However, the TCM treatment for COPD is mostly based on personal clinical experiences and lacks evidence-based support. In view of the above deficiency, more relevant researches have been conducted, which have obtained some certain achievements. Evidences indicate that TCM has some positive effects in the treatment of stable COPD, which could be showed in clinical trials [10, 12] and animal experiments [13–15]. It could bring a reduction of clinical symptoms and disease progression. Various clinical researches show that the combination of TCM and Western medicine can achieve better efficacy than Western medicine or TCM alone [16–18]. In summary, Western medicine, TCM, and combined treatment of both are all effective for COPD, and the combined treatment may have better efficacy.

With more and more different treatments being applied and proved efficacy in stable COPD patients, it is urgent to develop economic evaluation method or model to assess various treatments so that proper treatment could be chosen for patients and health policy-making department. More and more domestic scholars denote themselves into the work of health economics evaluation of COPD and try their best to find out the optimal treatment under China's conditions. COPD is a chronic disease with many different states, so in the research of health economics evaluation of prevention and treatments, the number of patients, cost, and utility in every disease state reduced by TCM will determine the accuracy of CUA. At present, decision tree model is used in most literature, but large deviation existed, so a new evaluation model needs to be developed.

Markov model, a mathematical model named after the Russian mathematician Markov, is a tool for studying the “states” and “transition between states” in a system and can simulate the process of random events. With the present known conditions, the future evolution does not depend on the past states. The relative independence between “future” and “past” is called Markov characteristics, and the random process is called Markov process. In the Markov process, the state obtained by the Nth time transition just depends on the result of the previous transition. Because of its characteristics of relative independence between different states, Markov model can be used in health economics evaluation of COPD.

This paper establishes a health economics evaluation model of different interventions on chronic diseases and conducts the health economics evaluation of different interventions on stable COPD based on Markov model.

## 2. Subjects and Methods

### 2.1. Data Resources

The data used in this study came from 4 subcenters of a key scientific and technological project of Henan Province named “Comparative Benefit Study of Three Different Treatments for Stable COPD Patients” (No. 132101510003) and was authorized by the project leader Professor Jian-sheng Li. The study has been registered on NIH (Trial ID=NCT01836016) and published in Trials [20]. The study is a multicenter, pragmatic, randomized, controlled trial, with a 26-week treatment period and a 26-week follow-up period. Patients who participated in the trail were aged from 18 to 80 years, as COPD in stable stage from mild to very severe, with given TCM syndrome pattern criteria, such as lung qi deficiency, lung-spleen qi deficiency, lung-kidney qi deficiency, or lung-kidney qi and yin deficiency, and they did not participate in other clinical trials one month before the run-in period and were entitled to medical informed consent. Patients who are pregnant or nursing and who suffered from any mental disorder and unable to understand the study were excluded from the trial, and so does the one who has underwent resection, radiotherapy, and chemotherapy in the past five years or was diagnosed with other respiratory disorders instead of COPD, such as bronchiectasis, tuberculosis, pulmonary fibrosis, or pulmonary thromboembolic or suffered from other diseases like concurrent heart failure (Grade III or above), myocardial infarction within six months, or concurrent serious hepatic and renal diseases. A stratified and block randomization design has been adopted. A total of 360 patients from 6 centers have been enrolled and randomly allocated to one of the three different treatment groups, Western medicine group, TCM group, and combined group. Patients in the Western medicine group have been divided into 4 groups and then were prescribed Salbutamol, Formoterol, and Salmeterol/fluticasone, respectively. Patients in TCM group have been treated with Chinese herbal medicine, Bufei granule, Bu-Fei Jian-Pi granule, Bu-Fei Yi-Shen granule, and Yi-Qi Zi-Shen granule. Patients in combined group have received the intervention of both Western and TCM medicine at the same time. Primary outcome of the study is exacerbation of COPD, with the secondary outcome being lung function, dyspnea, 6-minute walking distance, quality of life, and economic evaluation. Besides the original clinical data, the relevant economic indicators, such as cost, effectiveness, and utility, have been calculated in this paper. Cost-effectiveness analysis (CEA) has been applied to calculating the costs of reduction of AE, and the CUA to calculating the incremental costs per QALY.

### 2.2. Establishment of Decision Tree Markov Model

Decision tree Markov model has been established by TreeAge Pro 2011 (ID 1.0.12.1-v20110119). The process is to establish Markov model first and then to set up the decision tree model. Markov model has been used to simulate the natural history of stable COPD and also to calculate the expected value of different treatments. Decision tree Markov model used in this paper is a static, definite, integrated, and closed model.

#### 2.2.1. Establishment of Markov Model

Markov model consists of four components, namely, Markov health state, transition cycle, transition probability, and health utility value. The procedure to establish the model follows the steps: (1) establishing Markov states and their mutual transformations; (2) ascertaining cycle periods and transition probabilities; (3) obtaining the costs and utilities of every state; (4) reckoning the costs and utilities of disease development process; (5) calculating the health economics indexes.

The bases of establishing Markov model of natural history for stable COPD are as follows: (1) GOLD 2018 Report; (2) guidelines of diagnosis and treatment for COPD patients formulated by CTS COPD group in 2013; (3) a systemic review of literature about natural history for stable COPD patients since 2001.

**Figure 1:**
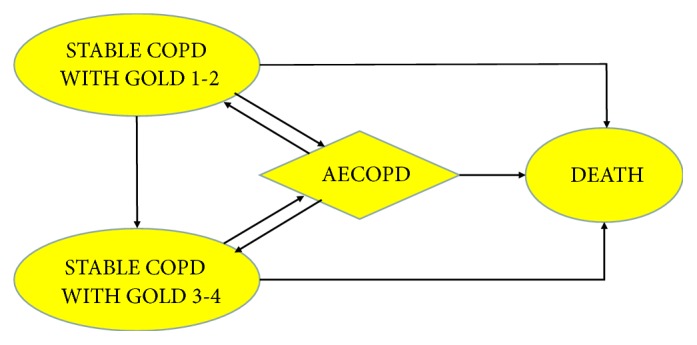
*Nature Course of Stable COPD*. It is the reference process for calculation, through which we also evaluate the health economics of COPD.

**Figure 2:**
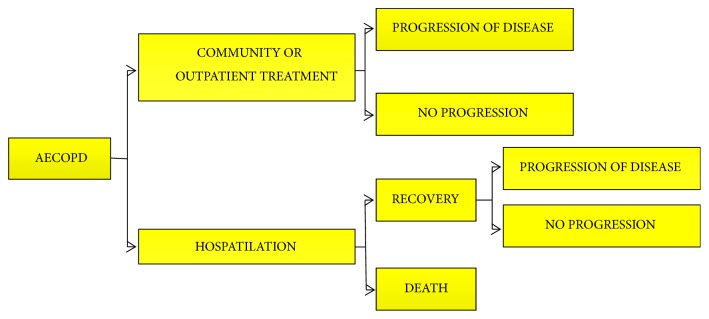
*Nature Course of AECOPD Prognosis*. Because AECOPD usually lasts for half a month and a cycle of Markov model in this study is one year, we cannot regard AE as a disease state but only a transient transition in case that the result will be biased. It is also the reference process for calculation.

The frame diagram of the Markov model is completed and shown in Figure 1. Because AECOPD usually lasts for half a month and a cycle of Markov model in this study is one year, we cannot regard AE as a disease state but only a transient transition to avoid the fact that the result is biased.  Figure 2 displayed the transition process of AECOPD. The Markov model has been defined with three Markov States, namely, GOLD1-2, GOLD3-4, and death.

#### 2.2.2. Establishment of Decision Tree

**Figure 3:**
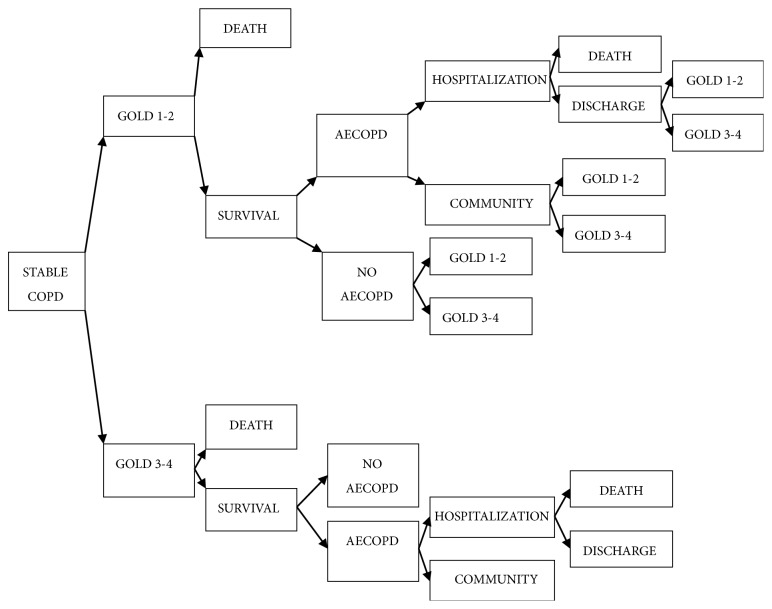
*Diagram of Decision Tree Markov Model for Each Treatment*. Referring to the nature course of stable COPD and AECOPD, decision tree Markov model has been established to conduct the research. The Markov model has been defined with three Markov States, namely, GOLD1-2, GOLD3-4, and death. Each state in the model transfers to other states or itself in a certain probability each year according to the arrow direction in the graph.

As shown in Figure 3, Markov decision tree model has been established to compare the costs and effectiveness among Western medicine, TCM, and combined group by TreeAge Pro 2011. A variety of parameters, including transition probabilities, costs, and utilities, were collected from the following ways:

1. First is making the best of the relevant data of the study.
2. The parameters less affected by regions and races were estimated by a systematic review of the literature newly published at home and abroad.
3. Some important parameters were important but difficult to determinate, such as the transition probabilities between different states; thus they were estimated by mathematical models.
4. One special investigation was conducted to obtain the direct medical and nonmedical costs of stable COPD patients in China.
5. Another special investigation was conducted to obtain the utility value of health states in stable COPD patients in China.
6. The discount rate, age, gender, mortality, and other parameters are based on the statistical data published by domestic authorities, such as various statistical yearbooks, National Health Yearbook survey, etc.

### 2.3. Cost-Utility Analysis

Economic evaluation index of CUA is CUR. CUR can reflect the costs of unit utility, whose formula is expressed as follows:

(1) $$\begin{array}{c} CUR=\frac{C}{U} \end{array}$$

ICUR is used as the final evaluation index to compare the two different treatments, and the formula is expressed as follows:

(2) $$\begin{array}{c} ICUR=\frac{\left(cost\;\;of\;\;A-cost\;\;of\;\;B\right)}{\left(QALYs\;\;of\;\;A-QALYs\;\;of\;\;B\right)}=\frac{\Delta C}{\Delta U} \end{array}$$

In this paper, when ICUR is calculated, utility is evaluated by QALY, and only direct cost is taken into consideration. The unit of ICUR is cost of per incremental QALY.

#### 2.3.1. Evaluation of Costs

The total costs included direct costs, indirect costs, and intangible costs. In view of the limited data collected in the supported project, this paper only calculated the direct cost. The direct costs of COPD patients included all kinds of costs directly spent in the process of diagnosis, treatment, and rehabilitation, such as various medical examinations, hospitalizations, treatment, and nursing. It also included other related expenses paid by patients in the process of receiving health services, such as expenses of nutrition, travel, purchasing rehabilitation products caused by COPD, and so on. Because the three different treatments for stable COPD patients were all safe, costs caused by side effects of medicine were not taken into account.

Outpatient visit, hospitalization, self-purchased drugs, rehabilitation, and other related medical expenses, such as oxygen therapy, were attributed to the medical costs for stable COPD patients, as the nonmedical costs included travel and nutrition for outpatient and hospitalization, while the same items were evaluated in AECOPD patients with different costs in every item.

#### 2.3.2. Calculation of Utilities

QALYs have been adopted in the calculation of utility in this study, which is the most commonly used index to represent the health utilities in CUA. It evaluated life expectancy combined with quality of life. Value of life expectancy could be got through life table, while quality of life was calculated referring to the Short Form-36 Health Survey Questionnaire (SF-36) score.

Quality of life had also been converted into index of life quality referring to criterion that utility value of full health was 1, with death being 0. QALYs would finally be obtained by converting life expectancy through index of life quality. For example, if the quality of life index was 1, one-year survival would be recorded as 1 QALY.

In this study, the SF-36 had been used to measure the utility value of stable COPD patients [21]. Ren XS et al. translated the SF-36 into Chinese referring to International Quality of Life Assessment (IQOLA) [22]. Wang XS et al. had measured 216 patients from two cancer medicine centers in China. The results showed that the alpha coefficients of the eight subscales ranged from 0.78 to 0.94, which reflected the stability of the Chinese version, and indicated that the SF-36 scale was suitable for Chinese people [23]. The score obtained through SF-36 could not be directly used to calculate QALYs. We should convert the data of SF-36 into SF-6D data first. And then, the UK SF-6D utility model would be applied to completing the conversion [24]. The details of UK SF-6D utility model could be got in Table 1. Then, to eliminate the interference of baseline utilities, analysis of covariance has been applied in the comparison for utility values obtained in each group after treatment.

### 2.4. Sensitivity Analysis

There are many uncertain factors in pharmacoeconomic evaluation, which could be determined by sensitivity analysis. We would confirm whether the final result varies with the change of parameters within a certain range. The process would be achieved by sensitivity analysis.

Univariate sensitivity analysis is used to analyze the link between parameters and experimental results when only one parameter in the range of preset sensitivity is changed each time while the other parameters are fixed. Similarly, the numbers of changed parameters are two and more for bivariate and multivariable sensitivity analysis.

### 2.5. Discount

Discount is the process of converting the future cost into the equivalent amount of present cost. The conversion ratio is called discount rate, which is usually expressed by symbol *i*. The costs of future and present are not equal but equivalent. When the intervention lasts for less than 1 year, the time value of the funds, namely, discount, can usually be ignored. But, when the intervention lasts for more than 1 year, discount must be taken into account. From now on, if the future costs at the end of the next 1,2, 3,…, n years are F_1_, F_2_, F_3_,…, F_n_ in turn, and P_n_ is the present value of F_n_, the formula for calculating the sum of all the present costs is showed as follows:

(3) $$\begin{array}{c} P=\overset{n}{\underset{t=1}{\sum }}P_{t}=\overset{n}{\underset{t=1}{\sum }}F_{t}{\left(\;1+i\;\right)}^{-t} \end{array}$$

In this paper, decision tree Markov model was used to predict the costs and utilities of patients in 40 years, so discount should be taken into consideration. The discount rate recommended by the* China Guidelines for Pharmacoeconomic Evaluations *(2011) was “one-year state-recommended interest rates or treasury rates” [25], and the guidelines also pointed out that “the sensitivity analysis of discount rate should be conducted and the recommended discount rate ranges from 0 to 8%.” In view of this, the baseline value of discount rate in this paper is 5%.

## 3. Results

### 3.1. General Characteristics

A total of 236 patients from four subcenters of the project, namely, First Affiliated Hospital of Henan University of Chinese Medicine, Shuguang Hospital Affiliated to Shanghai University of Traditional Chinese Medicine, Second Affiliated Hospital of Liaoning University of Traditional Chinese Medicine, and Shaanxi Hospital of Traditional Chinese Medicine, were brought into this study, with 79 cases in Western medicine group, 79 cases in TCM group, and 78 cases in combined group. The data of patients before treatment and three months and six months after treatment were adopted. As stated in Table 2, the results showed that no statistical difference among the three groups was found before treatment in age, gender, nationality, occupation types, education levels, and lung function.

### 3.2. Model Parameters

#### 3.2.1. Costs Parameters

The costs in stable period and AE of the three groups are shown in Table 3.

#### 3.2.2. Utility Parameters

The utility values of stable COPD patients in this paper come from the supported projects, which could be found in Table 4, without available data for AECOPD patients. According to the literature report [26, 27], we put forward the following assumptions: (1) medical interventions, including hospitalizations with severe attack and outpatient visits with mild attack, would be conducted for AECOPD patients; (2)* the utilities of patients with severe AECOPD (inpatients) were 30*%* of their stable stage, and patients with the mild AE (outpatients) were 85*%. The results of statistical analysis showed that the utilities of combined group had a more upward trend than TCM and Western medicine group with no statistical significance, as shown in Tables 5 and 6. The possible reason is that the sample size is too small. So, the CUA should be conducted to manage the further evaluation for the three different treatments.

#### 3.2.3. Transition Probability Parameters

There are only two options, namely, hospitalization and outpatient/community treatment, for patients with AECOPD. As the literature recorded, the mortalities of patients with GOLD 1 and GOLD 2 were not much different from that of the general population. The average age of the subjects was 64 years or so. The overall mortality of the whole population was originated from the China Population and Employment Statistics Yearbook 2010.

The treatment period of the study was half a year, so the achieved probabilities of AE and state transition were semiannual probabilities, which had been converted into annual probabilities by formula (4) and formula (5) [19].

(4) P=1−e−rt

(5) r=−ln⁡1−p0t0

Among them, *P* is the transition probability in a cycle, *t* is the time of the cycle, and P_0_ and T_0_ are the probabilities in the original clinical trial and the time of cycle period, respectively.

The average age of the patients in this study was about 64 years, with an estimated fatality rate of 3.2% [28]. The transition probability between each state was estimated by querying literature and data and showed in Table 7.

### 3.3. Simulation Results of Markov Cohort in Each Group

The results of Markov cohort simulation in Western medicine, TCM, and combined group showed that the accumulative QALYs per 100 000 people in 40 years were 1 702 773, 1 616 797, and 1 709 668 years, respectively, and the accumulative costs were 13 582 138 466, 12 073 904 113, and 14 656 607 371 Yuan, respectively. The CURs of the three groups were RMB 87 235, 74 602, and 87 223 Yuan per QALY, respectively. Compared with the Western medicine group and the TCM group, ICURs of the combined group were 8 707 and 41 705 Yuan as against Western medicine group and TCM group, respectively. The results were also showed in Tables 8 and 9.

### 3.4. Sensitivity Analysis

**Figure 4:**
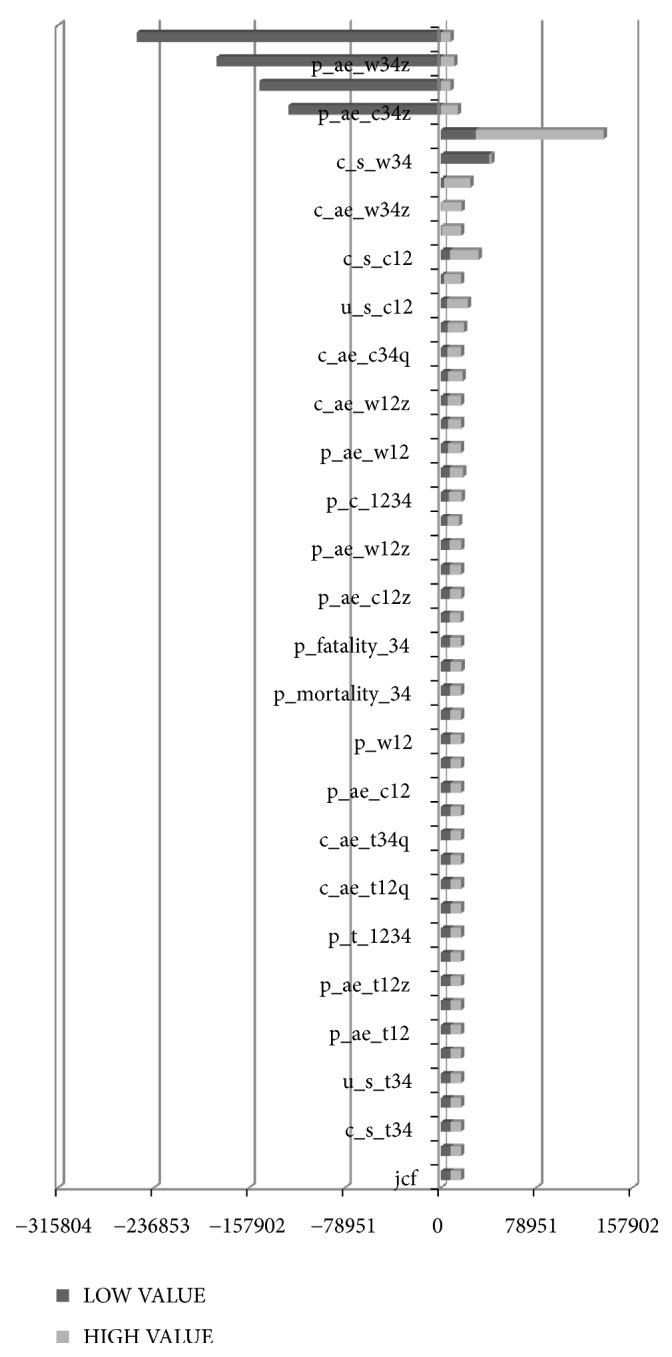
*Univariate sensitivity analysis between combined group and Western medicine group*. The results show that parameters vary independently within the range of sensitivity analysis except c_s_c34, p_ae_c34z, u_s_c34, p_ae_w34z, and u_s_w34, and the ICUR is less than the social willingness-to-pay all the time (1 time per capita GDP = 78 951.15 Yuan). With c_s_c34 fluctuating in its sensitivity analysis range, ICUR is less than two times of social willingness-to-pay all the time. With p_ae_c34z, u_s_c34, p_ae_w34z, and u_s_w34 fluctuating in the range of sensitivity analysis, ICUR will be less than zero if the utilities of combined group were lower than the Western medicine group.

**Figure 5:**
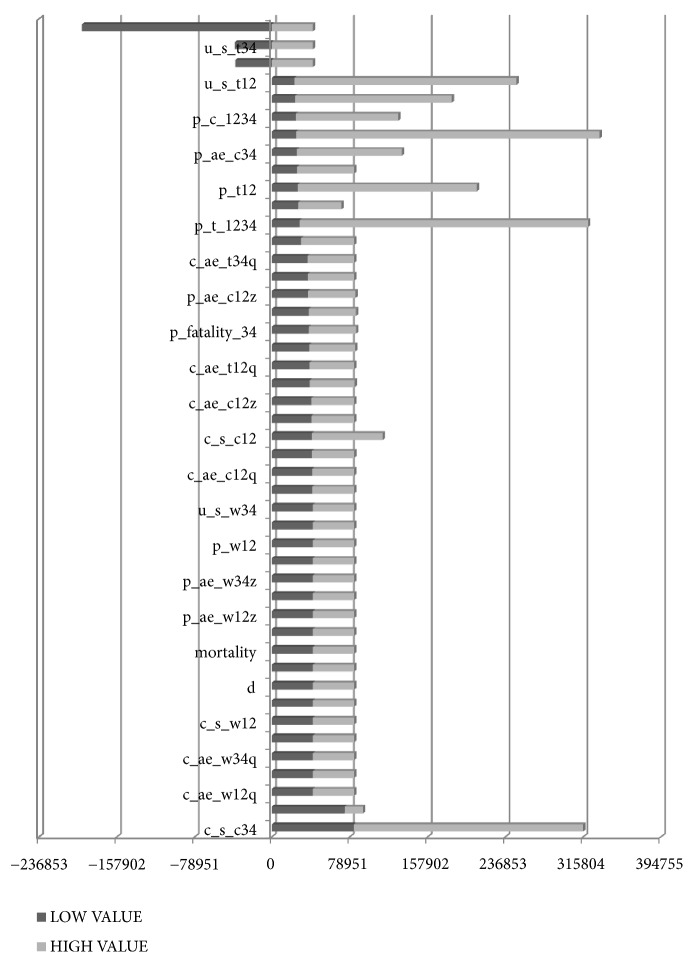
*Univariate sensitivity analysis between combined group and TCM group*. The results show that parameters vary independently within the range of sensitivity analysis except p_t12, p_t1234, p_ae_c34z, p_ae_t34z, u_s_t34, u_s_c34, u_s_c12, u_s_t12, p_c12, and c_s_c34, and the ICUR is always less than the social willingness-to-pay. With parameter p_t12 fluctuating in its sensitivity analysis range, ICUR is less than two times of social willingness-to-pay all the time. With u_s_t12, p_c12, p_t1234, and c_s_c34 fluctuating in the range of sensitivity analysis, the ICUR may exceed three times of social willingness-to-pay. With p_ae_c34z, p_ae_t34z, u_s_t34, and u_s_c34 fluctuating in the range of sensitivity analysis, ICUR will be less than zero if the utilities of combined group were lower than the TCM group.

Sensitivity analysis was conducted to observe the stability of baseline results by changing relevant parameters and the structure of the model and to determine their influence on the fitting results of the model at baseline. We would also find out the main factors that affect the model fitting results at baseline by sensitivity analysis. In this study, TreeAge Pro 2011 software was used to analyze the parameters that affect the operation of the model by univariate sensitivity analysis. The sensitivity analysis range of the cost parameters fluctuated 30% above and below the baseline value, with 20% fluctuation for utility and probability parameters. The results of sensitivity analysis for the main parameters were shown in Figures 4 and 5. The results showed that the reliability of the results of this study was good, and the model ran stably except for the impact of a few parameters.

## 4. Discussion

### 4.1. Medical and Economic Effects of TCM in Treating Stable COPD Patients

COPD belongs to the “cough,” “asthma,” and “pulmonary distension” in TCM, suffering from chronic cough and asthma, with prolonged course and recurrent attacks. It was first recorded in Lingshu more than 2000 years ago. In the stable period of COPD, we can improve positive qi, relieve symptoms, and reduce the incidence of disease by comprehensive therapy, as was recorded in Suwen. In recent years, great achievement has been made in the clinical research in the treatment of stable COPD patients by TCM. Various studies in the treatment of stable COPD show that TCM can bring a reduction of AE and improve the clinical symptoms, exercise capacity, lung function, and quality of life [29–31]. We can see that the clinical effect of TCM in the treatment for stable COPD patients is obvious, which is the basis of health economics evaluation. Since the costs of TCM have risen substantially in recent years, it is very important to evaluate its health economics too.

This study belongs to the comparative effectiveness research on COPD. Comparative effectiveness research is a new concept being put forward in recent years, through which we can determine the best intervention or strategy to provide reference for translational medicine and formulating health policies by comparing the benefits and risks of different interventions or clinical strategies [32]. At present, there are few studies about cost-effectiveness of COPD [33]. The well-known TORCH and UPLIFT tests have made a comparative study of salmeterol, fluticasone, salmeterol/fluticasone, and tiotropium bromide [6, 7, 28], and low-dose sustained-release theophylline tablet and carbocisteine tablet were also studied in China [9, 34]. However, there are few reports about research on comparative effectiveness between three or more drugs.

This study is a comparative effectiveness research on the health economics evaluation of stable COPD patients under the intervention of three different treatments based on Markov model, which will make up for the gaps in the comparative benefit research of TCM to a certain extent.

### 4.2. Using COPD as an Example to Explore the Assessment Method of Disease Burden Based on Markov Decision Model under the Intervention of TCM

At present, the evaluation studies of TCM intervention on chronic diseases mainly focus on efficacy and safety, ignoring the evaluation of disease burden and health economics, especially the scientific evaluation methods or models. With the improvement of people's health awareness and the emergence of scientific evidence to confirm the efficacy of TCM, more and more patients, especially those with chronic diseases, begin to choose TCM. But the prices of raw materials and treatment costs have increased with the increasing demand for TCM services. With the increasing morbidity of chronic diseases, an increasing economic burden has appeared for the nation, society, and families. The application of TCM is quite limited due to the absence of evaluation methods of disease burden; therefore it is urgent to establish an evaluation method or model of disease burden for TCM in the treatment of chronic disease.

So far, the commonly used health economics evaluation methods for COPD prevention strategies include cost-minimization analysis, CBA (cost-benefit analysis), CEA, and CUA.

CBA is the most basic and earliest evaluation method used in pharmacoeconomics study and plays an important role in the rise and development of pharmacoeconomics. Its aim is to convert the ultimate treatment effect, such as reducing disability rates and saving lives, into money, so that we can compare output and input directly to reveal different economic values of different treatments. However, there are many problems in this method such as that we could not accurately measure the benefit values and whether it is ethical to convert the value of people into money [35].

The core of CEA is the incremental cost-effectiveness ratio (ICER), which measures the different costs for a unit of additional benefit gained from a new treatment [36]. Because of its mature theory and clear assessment method, CEA had become the most commonly used method in health economic research. With different measurement units in different researches, the evaluation between different researches is difficult to be completed by CEA, and the improvement of patients' quality of life is not taken into consideration.

In view of above problems, CUA was born. CUA is a special type of CEA, measuring the quantity and quality of health output at the same time, and it is also the most commonly used method in the field of international pharmacoeconomics. The common thing between CEA and CUA lies in the measurement of costs while their difference is the measurement of health output. This method converts patient's quality of life into quality-adjusted life year (QALY) or disability-adjusted life year (DALY), and then the cost of per QALY would be calculated. In terms of output, CUA evaluates not only the effect of treatment on patient's survival time but also patient's quality of life. Therefore, this evaluation method would be used in wilder area than other methods, especially in the economic evaluation of treatment in chronic disease.

With long course, complexity, and dynamic changes, the processes and development outcomes of chronic diseases are difficult to predict. As a result, it is also difficult to conduct the disease burden evaluation by traditional methods of health economics evaluation, such as cost-minimization analysis, CBA, CEA, and CUA. At the same time, more and more evaluation models are being used in health economics evaluation of COPD prevention strategies, such as decision tree model, Markov model, and discrete choice model. And the most commonly used models are the first two.

Decision tree model is one of the most mature decision analysis models at present. It is characterized by the fact that the problem to be solved can be structured. However, the events in decision tree model are deemed to happen in the instantaneous discrete times. Unless the analyst specializes the different branches of decision tree, there is no clear and definite time limitation. Moreover, decision tree models will become very complex when applied to complex, long-term predictions, especially for chronic diseases. Many persistent risk factors for chronic disease, such as AE and death risk, have two important characteristics. First, the time when the risk will happen is uncertain. Second, a given risk may happen more than once. In this case, if the decision tree model were used, many mutually exclusive paths in the decision tree would become quite complex, which will bring great inconvenience into the analysis.

By contrast, the superiority of Markov model emerges. Markov model is a model for random processes. It divides the disease process into several different health states (Markov states). Considering the development processes simulated through the transition probabilities of every state in a certain period of time (Markov cycle), and the health utility value and resource consumption in each state, the outcomes and costs of disease development could be estimated by multiple cycle operations [37]. According to whether the transition probability is constant, Markov model is divided into Markov chain and time-dependent Markov process. Based on time and state, it would be divided into four types: time continuous, state discrete, time discrete, and state continuous. And there is also a cross between the above classification criteria. Because the transitions between various states in the processes of chronic diseases change over time, Markov model with continuous time and discrete state is more commonly used in the study of chronic disease [38], which is also the method applied in this paper.

### 4.3. Cost-Utility Analysis of Three Different Treatments for Stable COPD Patients

Because of the broad applicability of its health output index, CUA is more valuable to health policy makers than CEA. Researchers will get more reliable and applicable results under the following conditions [19]: ①health related quality of life was the important output index; ②health related quality of life was one of the important output indexes; ③the program affected both morbidity and mortality, and researchers intended to utilizing a universal measurement unit to take their impact into account; ④compared to a wide range of various results, a universal measurement unit as a contrast indicator would be applied; ⑤the research was conducted to be compared to that evaluated with CUA; ⑥the research goal was to optimize the allocation of limited health resources with considering all possible options and to maximize health outcomes. This study focused on the economic benefits of three different treatments for improving the quality of patients' life, so CUA would be the more suitable analysis method.

In the real-world research of health decision-making, costs, equality, ethics, and other factors of health should be taken into consideration, so this paper quoted external reference value, namely, the threshold criteria of pharmacoeconomic evaluation, to synthesize the abovementioned factors to conduct comprehensive evaluation of the three different treatments. If the incremental costs of a treatment exceeded the threshold for incremental unit effect, promotion and application were not recommended, and vice versa.

In incremental analysis, there is no uniform standard for QALY value in China, which generally refers to local GDP per capita. The average of GDP per capita in 4 cities was brought into study. Because this study has gone through 2013 and 2014, the GDP per capita of each city was the average of 2013 and 2014, and the threshold value of this paper with RMB 78 951.15 Yuan was determined.

According to the relationship between GDP per capita and ICUR, the threshold criteria for CEA recommended by the WHO are as follows [39]: first, ICUR less than 1 time of GDP per capita is very cost-effective; second, ICUR more than 1 time and less than 3 times of GDP per capita is cost-effective; third, ICUR more than 3 times of GDP per capita is not cost-effective. The threshold criteria for CEA of Pharmacoeconomics studies had not been published in China, so this paper referred to the WHO criteria.

This study focuses on health-related quality of life, so it is proper to apply CUA to managing the health economics evaluation. By using Markov cohort simulation, the results showed that the QALYs of every 100 000 people in Western medicine group, TCM group, and combined group in 40 years were 1 702 773, 1 616 797, and 1 709 668 years, and the costs were 13 582 138 466, 12 073 904 113, and 14 656 607 371 Yuan. The CURs of the three groups were 87 235, 74 602, and 87 223 Yuan/QALY, respectively. Compared with Western medicine group and TCM group, the costs of every incremental QALY in the combined group were 8 707 and 41 705 Yuan, respectively. As the results showed, the combined group may be more economic than the other two groups, and its ICUR was less than the social willingness-to-pay. Therefore, the combined treatment has a good prospect of promotion in terms of the current economic development level in China. In this paper, results of univariate sensitivity analysis showed that the reliability of the results of this study was good, and the model ran stably except for the impact of a few parameters.

### 4.4. Deficiencies of This Study

Markov model generally predicts the possible clinical outcomes and describes behaviors, predictions, and possible consequences through stochastic mathematical models. When Markov model is being established for TCM, because of its great individualization, standardization of intervention and processes of mathematical quantization will become difficult, and the random process and utility value will also be difficult to determinate at the same time. In the operation of model in this study, there are still some limitations on the determination and adoption of relevant parameters. Some unavailable parameters, which may influence the operation of model and accuracy of results, were estimated. And some relevant parameters may change over time, such as prices of medicine and medical service. If possible, latest and available data need to be got to update the operation of model. For COPD, if it was only based on the clinical outcomes observed and estimated in 236 cases, significant bias may arise in the occurrence time and incidence. In this paper, Markov cohort simulation study based on previous data offsets the impact of insufficient sample size on the results to some extent.

## 5. Conclusion

Compared with Western medicine and TCM group, combined treatment has a lower cost, higher health output, and more economic advantages in the long run. It is recommended to apply Markov model to conducting health economics evaluation of different treatments for COPD. As an exploratory study, this paper attempts to establish the evaluation method of three treatments for COPD based on Markov model, which can lay the foundation for further research and may also be used as an example to assess other chronic diseases.

## Figures and Tables

**Table 1:** UK SF-6D Score Model^*∗*^.

Whole Condition		Physical function		Role limitation		Social function		Pain		Mental health		Vitality	
Item	Score	Level	Score	Level	Score	Level	Score	Level	Score	Level	Score	Level	Score
C^±^	1	PF1	0	RL1	0	SF1	0	PAIN1	0	MH1	0	VIT1	0
MOST&	-0.070	PF2	-0.053	RL2	-0.053	SF2	-0.055	PAIN2	-0.047	MH2	-0.049	VIT2	-0.086
		PF3	-0.011	RL3	-0.055	SF3	-0.067	PAIN3	-0.025	MH3	-0.042	VIT3	-0.061
		PF4	-0.040	RL4	-0.050	SF4	-0.070	PAIN4	-0.056	MH4	-0.109	VIT4	-0.054
		PF5	-0.054			SF5	-0.087	PAIN5	-0.091	MH5	-0.128	VIT5	-0.091
		PF6	-0.111					PAIN6	-0.167				

^*∗*^Utility value =C+PF+RL+SF+PAIN+MH+VIT+MOST. The utility is between 0 and 1. ^±^C is a constant. &MOST will be used if any dimension is at the most critical level.

**Table 2:** General Characteristics of Patients at Baseline^#^.

Characteristics	Western medicine group	TCM group	Combined group	*χ* ^2^/*F*	P
Average age—yr^*∗*^	61.86 ± 9.82	64.01 ± 11.03	63.76 ± 10.30	1.009	0.366
Male sex — no. (%)	58(73.42)	53(67.09)	55(70.51)	0.684	0.760
Han nationality— no. (%)	74(93.67)	77(97.47)	77(97.47)	1.798	0.517&
Age levels—yr					
Under 40	3(3.80)	2(2.53)	3(3.85)	8.890	0.346&
40-	6(7.59)	8(10.13)	7(8.97)		
50-	21(26.58)	17(21.52)	17(21.79)		
60-	37(46.84)	27(34.18)	27(34.62)		
70-	12(15.19)	25(31.65)	24(30.77)		
Education levels					
Illiteracy/ Semiliterate — no. (%)	4(5.06)	3(3.80)	3(3.85)	7.614	0.673&
Primary school — no. (%)	11(13.92)	11(13.92)	10(12.82)		
Junior middle school — no. (%)	31(39.24)	28(35.44)	33(42.31)		
Senior middle school — no. (%)	20(25.32)	21(26.58)	24(30.77)		
Junior college — no. (%)	8(10.13)	12(15.19)	6(7.69)		
Undergraduate — no. (%)	5(6.33)	5(6.33)	2(2.56)		
Occupation					
Worker — no. (%)	5(6.33)	9(11.39)	5(6.41)	14.921	0.482&
Farmer — no. (%)	17(21.52)	11(13.92)	13(16.67)		
Intellectual — no. (%)	3(3.80)	2(2.53)	2(2.56)		
Manager — no. (%)	4(5.06)	3(3.80)	0(0.00)		
Service — no. (%)	2(2.53)	3(3.80)	0(0.00)		
Retirement — no. (%)	44(55.70)	49(62.03)	53(67.95)		
Unemployed— no. (%)	2(2.53)	2(2.53)	4(5.13)		
Laid-off— no. (%)	1(1.27)	0(0.00)	0(0.00)		
Others— no. (%)	1(1.27)	0(0.00)	1(1.28)		
Lung function					
GOLD 1-2— no.	45	43	39	0.784	0.676
GOLD 3-4 — no.	34	36	39		

^*∗*^Plus–minus values are means ± SD. ^#^There were no significant differences between the three groups in any of the baseline characteristics. & Fish's exact test.

**Table 3:** Annual Costs of COPD Patients^@^.

	GOLD 1-2			GOLD 3-4		
	Stable period	Mild attack (outpatient/Community)	Severe attack (Hospitalization)	Stable period	Mild attack (outpatient/Community)	Severe attack (Hospitalization)
Western medicine group	2998.36 ± 1750.01	7345.33 ± 10753.48	17882.67 ± 15426.19	5324.59 ± 985.55	9795.00 ± 17208.92	10735.00 ± 7861.86
TCM group	6829.00 ± 525.18	5974.76 ± 11563.94	12660.00 ± 16772.57	7015.94 ± 462.63	2567.78 ± 1170.40	8267.75 ± 7123.00
Combined group	9342.12 ± 1621.49	1597.50 ± 1610.06	8200 ± 282.84	11306.64 ± 770.09	2557.78 ± 1780.60	6605.72 ± 5450.84

^@^Plus–minus values are means ± SD.

**Table 4:** Utility Values of Patients with Stable COPD^$^.

	Pretherapy		After treatment for 6 months	
	GOLD 1-2	GOLD 3-4	GOLD 1-2	GOLD 3-4
Western treatment group	0.660 ± 0.099	0.657 ± 0.099	0.691 ± 0.123	0.678 ± 0.105
TCM group	0.679 ± 0.108	0.641 ± 0.103	0.692 ± 0.107	0.702 ± 0.084
Combined group	0.697 ± 0.074	0.705 ± 0.094	0.709 ± 0.102	0.713 ± 0.121

^$^Plus–minus values are means ± SD.

**Table 5:** Comparison of Utilities among Three Groups after Treatment^△^.

Groups	Utilities	F	P
Western medicine group	0.662519 ± 0.1055043	1.834	0.162
TCM group	0.676278 ± 0.1004063		
Combined group	0.708756 ± 0.1021848		

^△^Plus–minus values are means ± SD.

**Table 6:** Comparison of Utilities between Every Two Groups after Treatment.

Groups		Difference of Mean Values	Standard Error	*P*
Combined group	Western medicine group	0.028	0.015	0.063
Combined group	TCM group	0.015	0.015	0.304
TCM group	Western medicine group	0.012	0.015	0.393

**Table 7:** Annual Probabilities of AE and Transitions between States for COPD patients.

Parameters	Probability
probability of mild AE with GOLD 1-2 in Western medicine group	0.67
probability of severe AE with GOLD 1-2 in Western medicine group	0.33
probability of mild AE with GOLD 3-4 in Western medicine group	0.5
probability of severe AE with GOLD 3-4 in Western medicine group	0.5
probability of mild AE with GOLD 1-2 in TCM group	0.82
probability of severe AE with GOLD 1-2 in TCM group	0.18
probability of mild AE with GOLD 3-4 in TCM group	0.53
probability of severe AE with GOLD 3-4 in TCM group	0.47
probability of mild AE with GOLD 1-2 in combined group	0.67
probability of severe AE with GOLD 1-2 in combined group	0.33
probability of mild AE with GOLD 3-4 in combined group	0.53
probability of severe AE with GOLD 3-4 in combined group	0.47
fatality rate of patients with GOLD 3-4	0.032 [19]
mortality rates of patients with GOLD 1-2	0.0286^&^
mortality rates of patients with GOLD 3-4	0.126 [6]
Probability of transforming GOLD 1-2 into GOLD 3-4 in Western medicine group	0.215
Probability of transforming GOLD 1-2 into GOLD 3-4 in TCM group	0.170
Probability of transforming GOLD 1-2 into GOLD 3-4 in combined group	0.124

^&^Data are derived from assumption.

**Table 8:** Utilities and Costs of Every 100,000 Patients in 40 Years.

Utility and Cost	Western medicine group	TCM group	Combined group	D-value between combined and Western group	D-value between combined and TCM group
Life year—yr	1 702 773	1 616 797	1 709 668	6 895	92 567
QALY—yr	1 556 961	1 618 433	1 680 364	123 403	61 931
Total cost—Yuan	13 582 138 466	12 073 904 113	14 656 607 371	1 074 460 000	2 582 700 000

**Table 9:** ICUR between Three Groups (Yuan).

CUA	Combined group vs Western medicine group	Combined group vs TCM group
Cost of every incremental life year	155 833	27 810
Cost of every incremental QALY	8 707	41 705

## Data Availability

The data used to support the findings of this study are not available now because the subject which the project is based on has not yet been finalized.
